# Editorial: Role of the vestibular system in the perception of time and space

**DOI:** 10.3389/fnint.2022.1067425

**Published:** 2022-11-10

**Authors:** Pierre Denise, Laurence R. Harris, Gilles Clément

**Affiliations:** ^1^INSERM and University of Caen Normandy, Caen, France; ^2^Psychology Department, York University, Toronto, ON, Canada

**Keywords:** time perception and vestibular system, time perception, spatial cognition, weightlessness, hypergravity

The aim of this Research Topic was to address the influence of the vestibular system on time perception and spatial cognition, as well as how they are linked (Figure 1). Accurate time perception is required for higher level cognitive abilities, such as planning, decision making, communication, and effective coordination. Spatial cognition includes body motion, perception of the self, verticality, and distance perception combined with spatial learning and memory. Spatial cognition supports our capability for balance, orientation, and navigation in the terrestrial environment through integration of vestibular information into central multi-sensory processing. Since time perception is an inseparable part of spatial cognition, there is a growing interest in the role of vestibular inputs to subjective time. This Research Topic attracted a wide range of submissions across the spectrum of this theme. With this editorial, we intend to discuss the submitted contributions within the broader context of this growing field.

**Figure 1:**
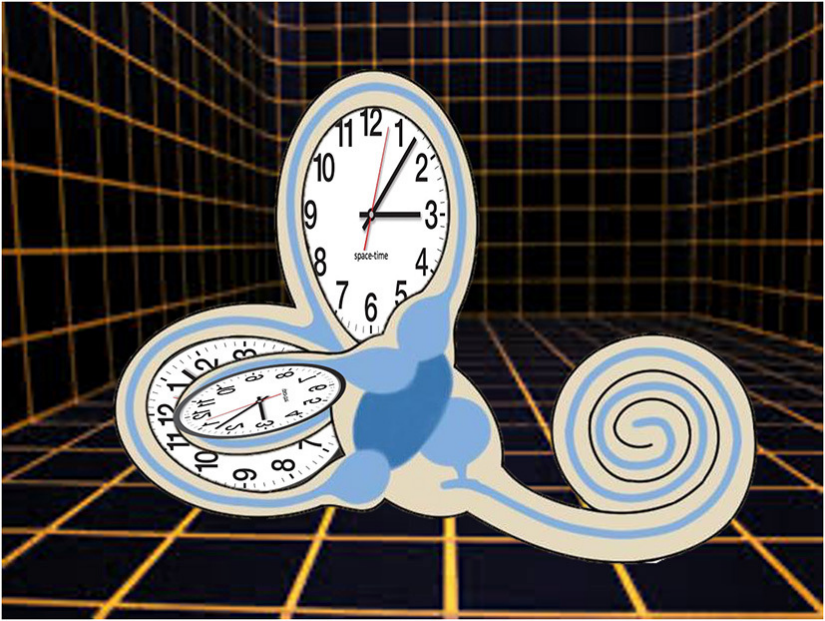
Artistic representation of the link between the perception of time and the vestibular system. Credit: Laurence Harris, York University.

Navigation, i.e., the ability to estimate our position and track and plan our path in our topographical environment, relies not only on detection and perception of our own motion but also on evaluation of the duration of this motion. Thus, it is no wonder that recent studies suggest that the representations of space and time share the same metrics and cortical network, presumably located in the right temporal-parietal junction (TPJ) (Delle Monache et al.). There is growing, but still scarce, evidence of links between spatial processing and time perception. For example, subjects who observe downscaled environments experience an underestimation of duration that is proportional to the scale-model environments being observed (Senna et al., 2021).

The vestibular system signals head movements and gravity, but its influence is not restricted to balance reflexes at the brainstem level as recent evidence shows that vestibular processing is involved in spatial cognition and time perception. Self-motion perception relies mainly on visual, vestibular, and somatosensory cues. In darkness, or in an impoverished environment in visual cues, the processing of vestibular cues is critical for spatial cognition (Stapel and Medendorp). However, the vestibular system signals head angular velocity (semicircular canals) and head linear accelerations, inertial or gravitational (otolith organs), while spatial orientation relies on position (angular and linear); thus, those vestibular signals need additional processing, i.e., time integration, in order to derive head angular position from head velocity and head linear position from head linear acceleration (Wagner et al.).

The TPJ continuously processes data from the visual, vestibular, and somatosensory channels for updating our spatial maps. However, the TPJ is also involved in time perception (Arshad et al.). As we know from experiments, patients with lesions of the TPJ display a correlated deficit in vestibular spatial perception and motion duration perception suggesting that temporal integration of self-motion velocity occurs in the TPJ and providing an explanation as to why a time perception deficit could lead to spatial disorientation (Utegaliyev et al.).

Changes in the level of gravity affect the vestibular system due to an increase or decrease in tonic otolith inputs. Therefore, alterations in time perception and space orientation are seen in subjects during whole body rotation (Alcantara-Thome et al.), head tilt (Bernard-Espina et al.), whole body tilt (Tekgün and Erdeniz), exposed to microgravity, and hypergravity (Clément, 2018), as well as in patients with mal de debarquement (Yakushin et al.) or vestibular disorders (Kwon et al.).

## Author contributions

All authors listed have made a substantial, direct, and intellectual contribution to the work and approved it for publication.

## Conflict of interest

The authors declare that the research was conducted in the absence of any commercial or financial relationships that could be construed as a potential conflict of interest.

## Publisher's note

All claims expressed in this article are solely those of the authors and do not necessarily represent those of their affiliated organizations, or those of the publisher, the editors and the reviewers. Any product that may be evaluated in this article, or claim that may be made by its manufacturer, is not guaranteed or endorsed by the publisher.
